# Insulators to Improve the Safety of Retroviral Vectors for HIV Gene Therapy

**DOI:** 10.3390/biomedicines4010004

**Published:** 2016-02-02

**Authors:** Diana L. Browning, Grant D. Trobridge

**Affiliations:** 1 School of Molecular Biosciences, Washington State University, Pullman, WA 99164, USA; dbrowning@vetmed.wsu.edu; 2 Pharmaceutical Sciences, College of Pharmacy, Washington State University Spokane, Spokane, WA 99202, USA

**Keywords:** retroviral vector, insulator, genotoxicity, insertional mutagenesis, anti-HIV, gene therapy, clinical trial

## Abstract

Retroviral vector gene therapy is a promising approach to treating HIV-1. However, integrated vectors are mutagens with the potential to dysregulate nearby genes and cause severe adverse side effects. Leukemia has already been a documented severe adverse event in gene therapy clinical trials for the treatment of primary immunodeficiencies. These side effects will need to be reduced or avoided if retroviral vectors are to be used clinically for HIV-1 treatment. The addition of chromatin insulators to retroviral vectors is a potential strategy for reducing adverse side effects. Insulators have already been effectively used in retroviral vectors to reduce genotoxicity in pre-clinical studies. Here, we will review how insulators function, genotoxicity in gene therapy clinical trials, the design of insulated retroviral vectors, promising results from insulated retroviral vector studies, and considerations for the development of insulated retroviral treatment vectors for HIV-1 gene therapy.

## 1. Introduction

Gene therapy is a promising alternative treatment option for HIV. Retroviral vectors are the favored method for effectively delivering anti-HIV genes to T cells or hematopoietic stem cells (HSC) for transplantation. This is in part because retroviral vectors integrate into the host genome, thereby permanently incorporating a therapeutic transgene into the host cell genome. A major advantage of integrating vectors over episomal vectors is that the integrated vector provirus with a therapeutic gene is efficiently transmitted to both daughter cells during division. Thus, during the massive expansion and differentiation of HSCs to produce the human immune system, the therapeutic transgene is transmitted to all mature white blood cells. This is also important for T cell therapies where there is expansion of gene-modified T cells. Unlike the parental wild-type viruses they are derived from, retroviral vectors have been significantly modified to improve safety and efficacy and are replication incompetent [1,2,3]. In current vector systems, the retrovirus enhancer and promoter elements have been removed from the long terminal repeats (LTR), and the encoded viral proteins have been extensively truncated, leaving only the minimal cis-acting sequences necessary for vector genome packaging and transduction. As such, the viral vector structural and enzymatic proteins are provided in trans for the purposes of vector production. Removal of viral genes prevents the potential pathogenic effects associated with the parent viruses, and also creates space for the addition of therapeutic gene cassettes. For anti-HIV therapy, gene expression could include any number of anti-HIV transgenes such as the membrane associated HIV fusion inhibitor C46, a trans-activation response element (TAR) RNA decoy, anti-HIV shRNAs or miRNA cassettes to inhibit HIV replication, or any combination thereof.

Several clinical trials have been completed or are underway to achieve the goal of gene-modified anti-HIV leukocytes in a patient’s blood using gene-modified T cells or HSCs. Both methods can utilize a variety of anti-HIV transgenes and so far both have encouraging clinical results [4,5,6,7,8,9]. However, the potential long-term effects of the treatment are substantially different for each method. Clinical studies using T cells have led to surprisingly long persistence of gene-modified T cells, but the number of long-term engrafted cells are expected to be lower than with stem cell transplantation [8,9,10]. Gene-modified T cells may provide transient clinical benefit without complete eradication of HIV-1 from the patient. However, without continued use of highly active antiretroviral therapy (HAART), HIV-1 infection can still reemerge. In contrast, HSC gene therapy allows for the continued production of gene-modified cells, including T cells [4,5,6], and mathematical modeling supports this concept [7,10]. Additionally, a stem cell gene therapy approach also allows for the anti-HIV gene to be expressed in all hematopoietic cells, reducing the potential for HIV-1 to find safe harbors for latency [11]. Therefore, the long-term safety of retroviral vectors for stem cell gene therapy is of great interest.

**Figure 1:**
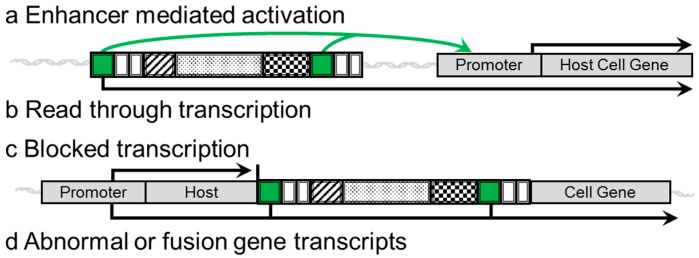
Retrovirus mediated mutagenesis. Proviruses can dysregulate host cell gene expression when integration occurs near a gene. (**a**) Enhancer elements within the retroviral vector can act on host cell promoters to increase expression; (**b**) Inefficient polyadenylation in the 3′ LTR leads to read-through transcription and increased expression of down-stream genes. Alternatively, integration within a host cell gene can cause aberrant expression by (**c**) promoting premature transcription termination or (**d**) forcing the formation of abnormal or viral/host cell gene transcripts. Green boxes denote the enhancer-promoter containing U3 of the retrovirus LTR and white boxes denote the R and U5 of the LTR. Hashed, plaid, and checkered boxes denote the retrovirus *gag*, *pol*, and *env* genes respectively.

The integration of retroviral vectors alters the chromosome such that retroviral vectors are de facto mutagens. The majority of vector integrations are benign, but an integration near or within a gene can lead to dysregulation of that gene. Depending on the gene, dysregulation can lead to tumorigenesis, specifically leukemia in HSC gene therapy [12,13,14,15,16,17]. The most commonly observed form of dysregulation by vector proviruses in clinical trials is enhancer-mediated activation of proto-oncogenes. For upregulation to occur, an integration event occurs near the proto-oncogene transcription start site, and an enhancer within the retroviral vector then activates the promoter (Figure 1a). Some retroviral vectors have inefficient transcription termination within the LTR such that transcription can continue through the 3′ LTR and into the chromosome (Figure 1b) [18]. This is known as read-through transcription. Integration within gene-coding regions can also lead to dysregulation by blocked transcription or abnormal fusion transcripts (Figure 1c,d).

A combination of approaches are being utilized to reduce vector-mediated dysregulation of host genes including the choice of retrovirus and the design of the vector, and are attempting to manipulate the integration profile of the vector [19,20,21,22]. The addition of the regulatory elements, known as insulators, may also be a promising approach to reducing enhancer-mediated activation [23]. Here, we discuss how insulators work, the design and promising results from insulated retroviral vector studies, and considerations for the development of insulated retroviral treatment vectors for HIV-1 gene therapy.

## 2. Insulators

**Figure 2:**
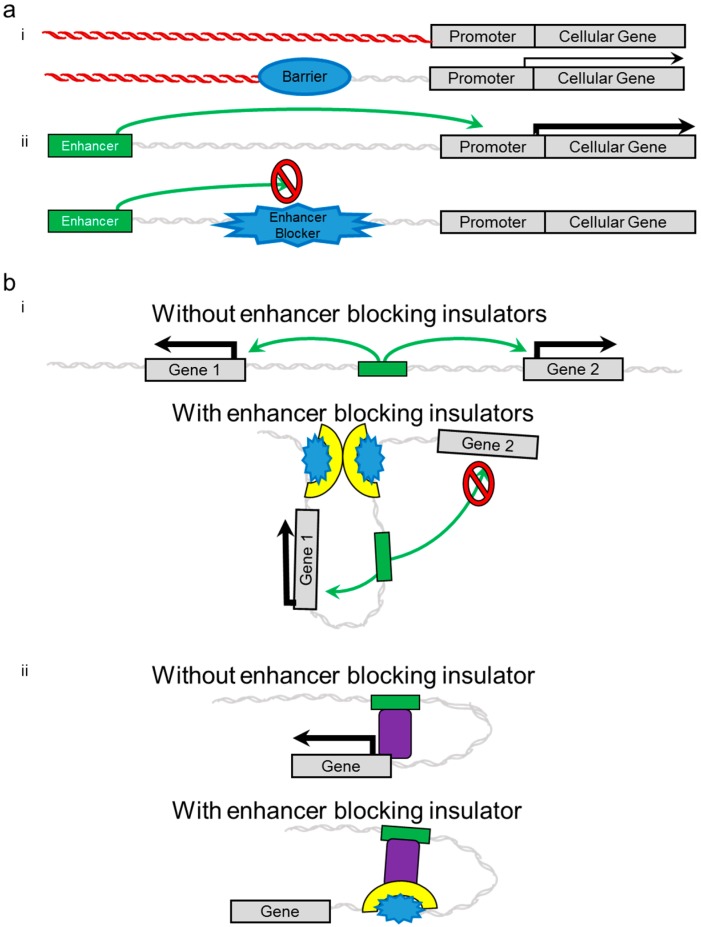
Insulator mechanisms of action. (**a**) Insulator elements recruit proteins that prevent inappropriate expression of genes. The barrier insulators (i) prevent promoter inactivation due to encroaching compact chromatin. The enhancer-blocking insulators (ii) prevent host cell gene promoters from being affected by nearby enhancers; (**b**) The enhancer-blocking insulators (blue stars) use multiple mechanisms to prevent host cell gene promoters from the influence of nearby enhancers. Prevention of enhancer (green rectangles) effects on promoters (grey rectangles) are achieved through (i) changing the chromatin architecture by forming domain loops where enhancers can only interact with promoters in the domain loop or (ii) by disrupting transcription factors (purple rectangle) recruited by enhancer elements. These actions require binding partners (yellow half circles) for insulating activity.

Originally described in the early 1990’s, insulators are genetic elements that protect promoters from their surrounding environment [24,25,26,27]. These elements contain binding sites for proteins that promote changes to chromatin structure in order to define domains of transcriptional activity. Insulators can be divided into two distinctive classes based on how they protect a promoter: barrier insulators and enhancer-blocking insulators (Figure 2a). Barrier insulators protect a promoter from becoming inactive due to encroaching compact chromatin [28]. Barrier insulators are found in the DNA where abrupt shifts from closed to open chromatin occur, thus allowing for transcription of genes within the area of open chromatin. In contrast to barrier insulators, the enhancer-blocking insulators function to prevent aberrant expression of promoters. An enhancer-blocking insulator prevents the enhancer from acting on a promoter when positioned between them. This activity is bidirectional, though some insulators have a greater effect in one direction than the other [29,30]. Enhancer-blocking insulators are of primary interest to increase the safety of retroviral vector gene therapy.

Enhancer-blocking insulator elements are highly conserved across all eukaryotes and are necessary for appropriate gene expression throughout the entire genome. There are tens of thousands of insulators in the human genome. Insulators recruit proteins that change the organization of the genome in order to maintain appropriate gene expression. In lower eukaryotes, a few proteins have been identified which bind insulators and impart enhancer-blocking activity [27]. In humans and other higher eukaryotes, all of the currently recognized enhancer-blocking insulators bind the protein CCCTC binding factor (CTCF) [31]. CTCF, originally described as a transcriptional repressor, is a versatile 11-zinc finger protein with multiple potential functions. In addition to its role in insulation, CTCF has been implicated as a negative regulator of the MYC oncogene, a transcriptional activator of the beta-amyloid precursor protein, as well as a component of X inactivation [32,33,34,35].

The role of CTCF as a mediator of enhancer-blocking activity has been extensively investigated [36,37]. The first insulator found in higher eukaryotes was the 4th DNaseI hypersensitivity site upstream of the chicken β-globin locus, known as cHS4. Based primarily on investigations of this insulator, we now know that CTCF imparts insulating activity by interacting with another CTCF through additional protein binding partners and by changing the higher-level chromatin structure through looping (Figure 2b) [38,39]. During interphase, DNA is loosely compacted so that gene expression can occur while also allowing the DNA to fit into the nucleus. At this time, DNA is wrapped around histones forming nucleosomes and further coiled into what is known as the 30-nm fiber. The 30-nm fiber is further ordered by attachments to protein scaffolds, the nuclear matrix, and to itself through additional protein interactions. Areas between attachment points form loops, and when demarcated by CTCF based insulators, these loops can act to define regions of transcriptional activity [40]. How and when these interactions change as gene expression changes is not well understood and is probably dependent upon the binding of additional proteins directly to CTCF or in close proximity on the DNA [41,42]. Alternatively, insulators may also directly interact with transcription factors or enhancers to block activity. Whole genome ChIP-Seq analysis and more recent extensive scanning of the human genome have revealed thousands of potential CTCF based insulator sites [29,43,44]. Though exceptions exist, these sites contain a core 20-bp binding region thought to bind the middle fingers of the zinc finger protein. This core region is enough to impart insulating activity [31]. Variation of the 20-bp core sequence affects CTCF binding and may contribute to defining the differences between constitutively active insulators and cell type or signaling specific insulators. Additional contributions may come from the surrounding genome where additional zinc fingers may interact with the DNA to increase specificity or recruit additional binding partners [41,43,45]. Future characterization of these sites should provide insight on the different mechanisms of action responsible for insulation.

## 3. Evidence of Genotoxicity and Adverse Side Effects in Clinical Trials

Retroviral vector-mediated HSC gene therapy has already been utilized in numerous clinical trials for the treatment of primary immunodeficiencies, blood disorders, as well as HIV-1 [46,47]. Promising therapeutic benefit has been seen in many of these clinical trials. However, clonal outgrowth has occurred in several trials for primary immunodeficiencies, specifically X-linked severe combined immunodeficiency disease (SCID-X1), Wiskott-Aldrich syndrome (WAS), chronic granulomatous disease (CGD), and the hemoglobinopathy, β-thalassemia [12,17,48,49,50,51,52]. These genotoxic events have often led to adverse side effects that are attributable to insertional mutagenesis by the integration of the gene therapy vector (Table 1).

**Table 1:** Integrations associated with adverse events in HSC (hematopoietic stem cells) gene therapy clinical trials.

Clinical Trial	# Participants	# Adverse	Integration Associated with Mutagenesis				Reference
			Oncogene	Position	kbp to TSS	+/−	
SCID-X1	20	5	*LMO2*	1st intron	2.0	−	[12]
				Upstream	2.9	+	[12]
				2nd intron	10.6	−	[12]
				Upstream	35.0	−	[50]
			*BML1* *	Upstream	49.5	+	[12]
			*CCDN2*	Upstream	2.4	−	[12]
WAS^#^	10	7	*LMO2* **	Upstream	20.6	−	[17]
				Upstream	32.3	−	[17]
				Upstream	33.0	−	[17]
				Upstream	1.5	−	[17]
				1st intron	8.7	−	[17]
				1st intron ***	24.7	−	[17]
			*MN1*	2nd intron **	351.7	−	[17]
			*MDS1* **	2nd intron	299.5	−	[17]
CGD	17	3	*MDS1*	Downstream	NR	NR	[51,52]

* BML1 integration in same clone as 3rd LMO2 integration; ** other integrations near oncogenes also found; *** contribution to development of AML (acute myeloid leukemia) after treatment for ALL (acute leukocyte leukemia); # reported for 6 of 7 patients with adverse events; transcription start sight (TSS); orientation of provirus with respect to oncogene (+/−); not reported or information unavailable (NR)

### 3.1. SCID-X1

The genotoxic events observed in early SCID-X1 clinical trials led to worldwide recognition that genotoxicity is a major obstacle for retroviral HSC gene therapy. In two trials with gammaretroviral vectors, 5 of 20 enrolled patients developed T cell leukemia [12,50]. In these patients, blast cells contained integrations near the proto-oncogenes LIM domain only 2 (*LMO2*), *BMI1*, and cyclin D2 (*CCDN2*). Four of the five patients had integrations near the promoter of *LMO2* with one of those patients having a second integration near *BMI1*. It is worth noting that these patients were infants that did not receive a conditioning regimen. When older children were treated with HSC gene therapy, no adverse events were reported, though the success from treatment was limited [53,54].

### 3.2. WAS

More recently, long-term results from a gammaretroviral gene therapy trial for WAS have been reported. Results were initially promising, with only a single patient out of ten having an adverse side effect within two years of transplant [55]. As of five years post-transplant, seven of the 10 patients have developed T acute leukocyte leukemia (ALL) or acute myeloid leukemia (AML) [17]. Of the patient samples analyzed for common insertion sites related to the leukemia phenotype, integrations near *LMO2* were strongly represented in patients with ALL and near the proto-oncogenes meningioma 1 (*MN1*) and myelodysplasia syndrome 1 (*MDS1*) were represented in patients with AML. Interestingly, two patients developed ALL and then presented with AML during treatment for ALL. During this time the contributing ALL clone decreased in prevalence while either a *MDS1* integrant containing clone or a *MN1* integrant containing clone became dominant. The patient who developed *MDS1* dominant leukemia did not survive.

### 3.3. CGD

Unlike gene therapy for the previous two immune disorders, the majority of CGD clinical trials have resulted in limited and transient correction of the disorder without severe genotoxic side effects [46,51]. Unfortunately, in the few instances where correction of the disorder was observed, severe genotoxic side effects eventually occurred. In one gammaretroviral vector clinical trial, two patients initially had a promising therapeutic benefit from the treatment. Both patients were found to have dominant clones with activating insertions near *MDS1* as well as two other oncogenes. In the short term, these activating insertions gave the recipients a therapeutic boost that added to the vector-mediated correction of CGD [48]. However, silencing of the therapeutic transgene eventually occurred, while the cells with integrants near and within the *MDS1/EVI1* locus continued to expand [52]. Overexpression from this locus led to genomic instability and myelodysplasia. Of the afflicted patients, one died of sepsis, and the other was given a second stem cell transplant as treatment. In a more recent clinical trial, two patients presented with dominant clones containing integrations in *MDS1* as well. Only one of the two presented with genomic instability and myelodysplasia prior to a second allogeneic stem cell transplant. The second patient was also given an allogenic stem cell transplant, which eliminated the *MDS1* integrant containing clone, and potentially prevented malignant transformation [51].

### 3.4. β-Thalassemia

Gene therapy for β-thalassemia has been uniquely challenging due to the requirement for transduction of a relatively high percentage of stem cells. The use of gammaretroviral vectors has not been effective due to size constraints and potential interference between the vector LTR and the expression elements necessary for therapeutic levels of β-globin gene expression [56]. Lentiviral vectors have therefore been favored for therapeutic vector development and brought to clinical trials. In the first clinical trial with a lentiviral vector, a patient with a successful transplantation of gene-modified stem cells had a remarkable therapeutic benefit from the treatment. Assessment of this patient’s peripheral blood showed a dominant clone containing an activating integration in the third intron of the high mobility group AT-hook 2 (*HMGA2*) locus. In this patient, the clone was only dominant in the myeloid restricted lineage and increased gene expression was limited to erythrocytes [49]. The vector used in this trial is also the first vector with an insulator to be used in clinical trials. The insulator used was a tandem repeated CTCF binding element from cHS4, which was reduced to a single element in the dominant clone. The reduction to a single CTCF binding element may have allowed for increased interaction between the elements necessary for therapeutic expression of β-globin and the promoter of *HMGA2*. This demonstrates the need for thorough investigations of insulated vector design and development, which is further discussed in the next section.

Although the adverse effects that can occur during HSC gene therapy can be severe, often the only alternative treatment option is waiting for an allogeneic stem cell transplant. Not all gammaretroviral gene therapy trials have had adverse genotoxic side effects. To date, many patients have been treated in clinical trials for ADA-SCID with a high success rate and no reported adverse genotoxic side effects [46]. However, gene therapy with gammaretroviral vectors has a marked risk for the development of adverse events attributable to vector-mediated genotoxicity. More recently, gene therapy trials have utilized lentiviral vectors, which are significantly less genotoxic, and have a reduced potential for adverse side effects [16,18,57]. Taken together, the severe adverse events seen in clinical trials are dependent on the interplay between therapeutic gene cassettes and the surrounding genome. Reducing the interaction has the potential to significantly improve the safety of retroviral vector gene therapy.

## 4. Development of Insulated Vectors

**Figure 3:**
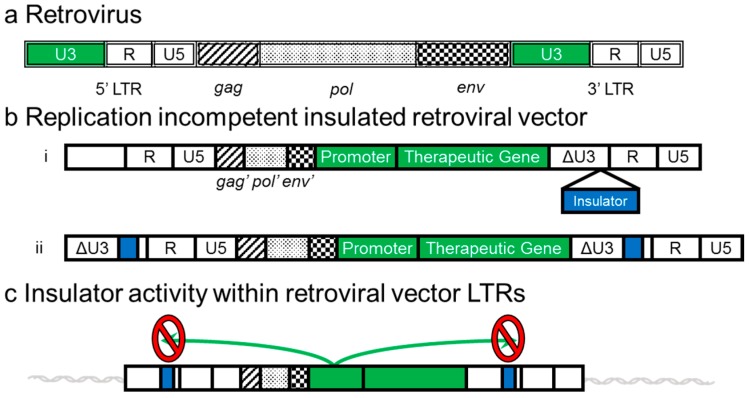
Development and design of insulated replication incompetent retroviral vectors. (**a**) A replication competent retrovirus (**b**) is significantly modified so that only the elements necessary for genome production, encapsidation, and integration are still intact. (i) The replication incompetent vector has the viral enhancer and promoter elements removed from the U3 of the 3′ long terminal repeat (ΔU3) and the 5′ U3 is either a viral promoter or been replaced by a stronger promoter for vector production. The insulator is positioned in the 3′ ΔU3. During reverse transcription the 3′ insulator containing ΔU3 is then transferred to the 5′ long terminal repeat replacing the promoter used for vector production. (ii) The final vector DNA genome ready for integration is thus flanked by insulators; (**c**) The insulators in the integrated vector provirus prevent the enhancer elements within the therapeutic gene cassette from acting on the surrounding host genome.

Though the specific mechanism of action is not fully understood, all known enhancer-blocking insulators function when positioned between an enhancer and a promoter. This defining characteristic must be taken into consideration for retroviral vector design in order to exploit these elements to increase safety. This presents the challenge of incorporating two copies of an insulator flanking the enhancer elements within the vector. The viral enhancer elements have been removed from the LTRs of current retroviral vectors. This leaves the internal enhancer-promoter of a therapeutic gene cassette as a potential source for enhancer-mediated activation of host genes [1,2,3]. Therefore, a successful insulated vector requires two insulators that flank the transgene cassette. The most popular insulated vector design features the insulator in the 3′ LTR U3. This utilizes the replication process of retroviral vectors wherein the U3 of the 3′ LTR is copied to the 5′ LTR and the U5 of the 5′ LTR is copied to the 3′ LTR during reverse transcription. This replication process results in an integrated vector provirus flanked with two identical insulators. This approach is potentially less susceptible to a recombination event during reverse transcription that would result in the loss of the therapeutic transgene cassette, which could occur if the transgene cassette was directly flanked by insulators [30,58]. Additionally, during vector development, enhancer sequences have been removed from the U3 of the vector LTRs without much effect on titer. This suggests a location where a new DNA element could be tolerated, essentially replacing one regulatory element with another (Figure 3).

In attempts to reduce the negative effects of insulators on vector production while maintaining or increasing insulation, the insulators themselves are being modified [37,42,59,60]. For example, the previously mentioned cHS4 is a commonly investigated insulator. The entire span of this insulator in the chicken genome is 1.2 kbp and has already been shown to have adverse effects on vector titers [59]. Through extensive assessment of the insulator, the most active portions of this insulator have been identified and much smaller versions containing the CTCF binding domain (250, 400, and 650 bp) developed for use in vectors [42,61,62]. Synthetic insulators with multiple identical copies of the CTCF binding domains have also been a popular strategy, though recombination leading to loss of the insulator is a concern [30,60,63]. As previously mentioned, this phenomena has already been observed in a β-thalassemia clinical trial where a tandem insulator was used in a lentiviral treatment vector. Prior to integration, one of the repeated insulators on each side was lost and potentially reduced the benefit of the insulator enough to allow the therapeutic gene expression elements to act on the surrounding genome. Recombination of direct repeats is common during transcription of an HIV vector when repeated sequences of 250 bp or more were in the middle of the virus [58]. We also found this to be true for repeated element insulators in the LTR of foamy viral vectors [30]. New designs of synthetic insulators should incorporate the more recently described CTCF insulators from the human genome to potentially avoid insulator failure due to recombination [44].

## 5. Insulated Retroviral Vectors

Insulators have now been evaluated in gammaretroviral, lentiviral, and most recently foamy viral vectors. At least one clinically relevant insulated vector has been developed for each of these vectors. Insulated vectors have had encouraging results in pre-clinical studies, suggesting their increased safety [16,30,57,64,65]. Despite the generally positive results, there are vector dependent insulator effects on titer as well as vector effects on insulator performance that could affect the use of insulated vectors in the clinic.

### 5.1. Gammaretroviral Vectors

Insulators have shown great promise in gammaretroviral vectors, in part due to the highly genotoxic nature of gammaretroviral vectors. The addition of insulators substantially decreases the genotoxic potential these vectors in *in vitro* genotoxicity assays [16]. However, the genotoxicity of insulated vectors is still readily measurable, limiting their clinical efficacy. The addition of a 1.2-kbp cHS4 insulator reduced the titer of a gammaretroviral vector threefold [66]. Reduced titers were also reported for insulated gammaretroviral vectors where a tandem repeat element insulator was used to directly flank the gene cassette [67].

### 5.2. Lentiviral Vectors

Lentiviral vectors are significantly less genotoxic than gammaretroviral vectors and, with the addition of insulators genotoxicity, becomes undetectable in *in vitro* genotoxicity assays [30,57]. To date the only reported pre-clinical test showing any level of measurable genotoxicity from an intact insulated lentiviral vector utilized tumor prone *Cdkn2a*^−/−^ mice, where the addition of an insulator modestly, though significantly increased the lifespan of these mice compared to uninsulated control vectors. Although, these mice still had reduced lifespans compared to no vector controls, the results show an added safety benefit to utilizing insulators in lentiviral vectors [65]. Insulator size has a strong negative influence on the titer of lentiviral vectors. This led to the development of the previously mentioned condensed cHS4 insulators, which are functional but not as effective as the full-length cHS4 [29,59,60]. Insulators with repeats of the active elements are susceptible to recombination in lentiviral vectors as well [49,63]. Interestingly, a full-length cHS4 insulated lentiviral based anti-HIV gene therapy vector has already been utilized in a preclinical study [68]. Despite the use of a large insulator, these vectors were produced at clinically relevant titers (all variants above 10^6^ IU/mL prior to concentration) and the vectors were successfully used to reduce HIV-1 infection in peripheral blood mononuclear cells. For these studies, the effects of the insulator on vector genotoxicity were not addressed.

### 5.3. Foamy Viral Vectors

Like lentiviral vectors, foamy viral vectors are also significantly less genotoxic than gammaretroviral vectors [18,30,69]. Foamy viral vectors have significantly reduced read-through transcription from the vector provirus than either gammaretroviral or lentiviral vectors [18], and promote the transformation of factor-dependent cell lines to factor-independent cells at a significantly lower frequency than gammaretroviral vectors [30]. Because of the already low genotoxic nature of foamy vectors, a rapid *in vitro* genotoxcity assay did not show a substantial impact of insulators on the safety of foamy viral vectors. However, our lab recently evaluated retroviral integration sites from insulated and uninsulated foamy viral vector-exposed human CD34^+^-enriched cord blood cells after *in vitro* culture for evidence of vector integration-mediated growth advantages. In the timeframe of the experiment, no individual retroviral integrations were identified as having a growth advantage. We did find that the presence of an insulator significantly reduced the accumulation of observed integrations found within 50,000 bp sized hotspots as early as five days post vector exposure. After an additional five days of culture, the number of insulated vector integrations within hotspots stayed the same while the integrations within hotspots of uninsulated foamy vector exposed cells increased [30]. The unchanged frequency at which integrated insulated vectors are observed within hotspots compared to the changed frequency of uninsulated vectors suggests that the insulators are reducing the effects of foamy virus integrations on the surrounding genome and potentially increasing the safety of these vectors. Future *in vivo* experiments will further clarify the effects of insulators on the safety of foamy viral vectors.

Similarly to other retroviral vectors, insulators can affect foamy vector titer. Interestingly, the degree of the affect is often orientation dependent [30]. A cHS4-based insulator reduced foamy vector titers five- to seven-fold in the forward orientation while only reducing titer threefold in the reverse orientation [30]. There are also insulators that do not affect foamy vector titer regardless of orientation [30]. Thus, insulated foamy vectors can be produced at high titers and appear to be promising for future clinical studies including anti-HIV gene therapy. Similar to lentiviral vectors, repeated element insulators are susceptible to recombination as well. The frequency of recombination is strongly influenced by the orientation of the repeated element insulator. We have found that these repeated element insulators have very low titers, which may be attributable to the frequency of recombination [30].

## 6. Considerations for the Development of Insulated Anti-HIV Retroviral Vectors

Though similar to designing retroviral gene therapy vectors for genetic disorders, the development of a retroviral vector to treat a transmissible retroviral disease presents a unique set of challenges and opportunities not seen for other gene therapy applications. In a traditional gene therapy setting, the choice of retroviral vector would primarily be a discussion of which vectors were the least genotoxic. However, for HIV-1 gene therapy, the effects of the therapeutic transgene on vector production must also be considered. Many anti-HIV therapeutic genes function by inhibiting HIV replication which may also inhibit retroviral vector production [70]. Reduced titers due to the anti-HIV transgene cassettes in lentiviral vectors have already been documented [70,71,72,73,74]. The risk of therapeutic vector/live virus recombination events is also another concern for HIV-derived vectors. Thus, foamy viral vectors have a major advantage in being less genotoxic than gammaretroviral vectors, and, unlike HIV-1 derived lentiviral vectors, foamy vector titers are not affected by anti-HIV transgenes [75,76].

## 7. Future Perspectives and Unique Opportunities for Anti-HIV Gene Therapy

The addition of insulators to anti-HIV retroviral gene therapy vectors is a very promising approach to reducing the potential genotoxicity of these vectors. Further reductions to genotoxicity may come by combining insulated vectors with other approaches currently being developed including transcriptional targeting [19] and integration site retargeting [77,78,79]. Unlike some gene therapy applications, where the therapeutic gene needs to be expressed in numerous cell types, anti-HIV therapy is targeted primarily to circulating T cells and macrophages. This provides a unique opportunity to also provide a tissue-specific mechanism to increase safety. By combining an insulator with a tissue-specific promoter, enhancer-mediated activity is typically substantially reduced and restricted to more mature cells, thus reducing the potential for negative effects on HSCs. Efforts to change or retarget the integration site preferences of retroviral vectors could also be used to further increase safety by promoting integration in genomic regions that are condensed in HSCs but open in mature circulating cells [77,78]. This would reduce the availability of actively transcribed host cell genes in proximity to vector integration sites in HSCs. Finally, retroviral anti-HIV gene therapy may be an ideal setting to utilize a suicide gene cassette. For this a cell death gene cassette, such as caspase 9, is added to the vector under the control of an inducible promoter. If an adverse side effect becomes apparent, such as the development of leukemia due to an outgrowth of an insertional mutagenesis event, a drug is administered to activate the inducible promoter in transduced cells. These cells would be induced to die, thus effectively eliminating the adverse effect [80,81,82,83,84]. At such a time, stem cell gene therapy can be repeated. Taken together, the development of safe and effective gene therapy retroviral vectors for HIV-1 gene therapy is extremely promising and insulators may be used in combination with other safety features.

## 8. Conclusions

Retroviral vector-mediated HSC gene therapy is a promising strategy for treating and effectively curing HIV-1 infected individuals. With these treatments comes a risk for the development of blood disorders from dysregulation of genes by the integrated vectors within the transduced stem cells. An addition of enhancer-blocking insulators to the retroviral vectors could reduce this risk and increase the safety of retroviral vectors for anti-HIV gene therapy.
